# A Q Method Approach to Evaluating Farmers’ Perceptions of Foot-and-Mouth Disease Vaccination in Vietnam

**DOI:** 10.3389/fvets.2017.00095

**Published:** 2017-06-26

**Authors:** Dinh Bao Truong, Aurélie Binot, Marisa Peyre, Ngoc Hai Nguyen, Stéphane Bertagnoli, Flavie Luce Goutard

**Affiliations:** ^1^UPR AGIRs Research Unit, Centre de Coopération Internationale en Recherche Agronomique pour le Développement (CIRAD), Montpellier, France; ^2^Faculty of Animal Science and Veterinary Medicine, Nong Lam University, Ho Chi Minh, Vietnam; ^3^Faculty Veterinary Medicine, Kasetsart University, Bangkok, Thailand; ^4^UMR INRA-ENVT IHAP, Université de Toulouse, Toulouse, France

**Keywords:** vaccination, farmers’ perceptions, foot-and-mouth disease, participatory methods, Q methodology, discourse

## Abstract

This study aims to explore the farmers’ perceptions of foot-and-mouth disease (FMD) vaccination using a reflexive research method called Q methodology. A structured sample was composed of 46 farmers selected according to gender, farming experience, level of education, and production type. Statements relevant to the farmers’ perceptions of and attitudes toward FMD vaccination, related to confidence, logistics, costs, and impacts of vaccination were developed. Results were analyzed by principal component analysis and factor analysis. The influence of demographics and characterized variables on the respondent’s contribution to each factor was also tested. Regarding the different beliefs and behavior toward FMD vaccination, the common perceptions held by Vietnamese cattle and pig farmers were divided into three discourses named Confidence (24 subjects), Belief (12 subjects), and Challenge (6 subjects). The identified discourses represented 57.3% of the variances. Consensus points were found, such as the feeling of being more secure after FMD vaccination campaigns; the fact that farmers take vaccination decisions themselves without being influenced by other stakeholders; the opinion that FMD vaccination is cheaper than the costs of treating a sick animal; and that vaccines provided by governmental authorities are of high quality. Part of the studied population did not consider vaccination to be the first choice strategy in prevention. This raises the question of how to improve the active participation of farmers in the FMD vaccine strategy. Taking into consideration farmers’ perceptions can help to implement feasible vaccination strategies at the local level.

## Introduction

Foot-and-mouth disease (FMD) is among the most widespread infectious diseases that harm the development of the world’s livestock sector (1). In order to tackle FMD outbreaks, various disease management approaches have been implemented in South-East Asia (SEA), including risk analysis, vaccination, surveillance networks, laboratory support, animal movement control, policy advocacy, support of private sector and other stakeholders, communication improvement between country members through workshops and meetings, and public awareness (2). Surveillance networks have been developed at national and also regional levels (e.g., South-East Asia and the China Foot-and-mouth Disease program, http://www.rr-asia.oie.int/activities/sub-regional-programme/stanz/seacfmd/). The efficiency of FMD surveillance and control programs in developing countries is often challenged by the issue of underreporting (3, 4). Owing to the low mortality rate, farmers often consider FMD as the second priority for control after haemorrhagic septicemia, despite its potential negative impact on production yield (3). However, FMD is known to cause significant financial losses for small producers and, therefore, to threaten the livelihood and food security of the poorest communities worldwide (4). For example, in Laos, it was estimated (in three provinces under study) that losses due to FMD varied from 381 to 1,124 US Dollars (USD) per household, per year, representing 16 to 60% of annual household income (5). In Vietnam, the annual average economic loss for each affected farm was estimated to be 84 USD for highland areas with low livestock density and up to 930 USD per farm for lowland areas with high livestock density (6). Moreover, a recent study on the financial impacts of swine diseases reported that the total cost of FMD was estimated to be 21.3, 23.8, and 27.8 USD per pig for a large farm, a fattening farm, and a smallholder, respectively (7). The financial impact of FMD on smallholder cattle farmers in southern Cambodia was estimated to range from 216 to 371 USD per animal, with an outbreak reducing annual household income by more than 11% (8). FMD also represents a major obstacle to international trade and a permanent risk to countries with an FMD-free status. For these reasons, FMD has been targeted by The World Organisation for Animal Health (OIE) and Food and Agriculture Organization (FAO) as a priority for disease control worldwide throughout a global strategy (1). Despite the availability of effective vaccines, the successful control of FMD remains very limited. The investments required to control the disease are substantial regarding financial and logistical resources (1).

In Vietnam, FMD is endemic with outbreaks occurring every year (4, 9, 10). Considering the importance of the disease, the Vietnam Ministry of Agriculture and Rural Development (MARD) has been implementing a national prevention and control program since 2006. This program is renewed every 5 years by the Department of Animal Health (DAH, subordinate of MARD)—which is in charge of disease surveillance at the central level. Some technical solutions are currently proposed in this program, such as the implementation of epidemiological and serological surveys, disease surveillance, animal movement control, vaccination, disinfection, awareness raising, and training workshops. Among these strategies, mass vaccination against FMD for all cattle and buffaloes within specific targeted areas is considered to be a valuable tool. According to the epidemiological situation, provinces of Vietnam are classified into two zones: high-risk (subdivided into control and buffer) and low-risk zones (11). The control zone (high-risk) consists of eight provinces along the northern border, six provinces along the southwest border, between Vietnam and Cambodia, and five provinces located on the border with Laos and the Central Highlands region. The buffer zone (high-risk) consists of 90 provinces adjacent to the control zone. The low-risk zone consists of nine provinces in the Red River Delta region, four important export provinces along the North Central Coast (Nghe An, Thanh Hoa) in the Red River delta region (Ninh Binh, Vinh Phuc), nine provinces in the Mekong Delta region, and three provinces in the South-East region and Ho Chi Minh City (11).

The surveillance and reporting system is mainly organized into three levels: (i) epidemiological unit of DAH at central level, (ii) epidemiological unit of Regional Office of Animal Health at regional level, epidemiological unit of sub-Department of Animal Health (sub-DAH) at province level, employees of the district office of animal health at intermediate level, and slaughters houses located in districts, and (iii) farmer, veterinary commune at local level (12). Three serotypes O, A, and Asia 1 have been detected in Vietnam (13, 14). According to information on the serotypes currently circulating in Vietnam, vaccines used in the field may be monovalent (serotype O) or bivalent (serotype O and A) (11, 15). The type of vaccine used varies every year according to the epidemiological situation of each location. For example, the sub-DAH of Long An province used a monovalent vaccine for pigs and cattle in 2012, but they had to switch to a bivalent vaccine in 2013 for cattle as serotype A was circulating at this time. The objective of the national program is to vaccinate 85 to 100% of the cattle and buffalo populations within the high-risk zones. In the low-risk zones, vaccination is only implemented in locations where an outbreak has been recorded by the provincial authority over the last 5 years. The main target animals for this program are cattle and buffaloes. The vaccination of pigs and other susceptible animals is not well-detailed in the program, and the decision is left to the sub-DAH. Vaccination is usually done twice yearly (March–April and September–October). Vaccination budgets for each zone are also different. In control and buffer zones, vaccine fees are financed up to 100 and 50% of their costs, respectively, by the national budget, while the labor cost of the commune’s veterinarian is paid for by the local authorities. In low-risk zones, these fees are paid for by the local authorities (11). The total estimated cost for the national program (national and local budget) for FMD prevention and control in Vietnam has recently been estimated at 36 million USD for the period of 2006–2010 and 32 million USD for the period of 2011–2015 (11). The following phase of the National Plan, from 2016 to 2020, has already been implemented in the field; it includes certain changes in the vaccination strategy for each zone and the creation of an animal identification system (15).

As previously described, the primary FMD prevention and control strategy in Vietnam is, therefore, to concentrate vaccination efforts within the “hot spots,” which are the zones identified with a higher risk of outbreaks. However, this strategy comes up against many logistic and economic constraints, and its effectiveness has yet to be proven regarding vaccine coverage and disease control (11, 15). The location of hot spots is not easy to estimate because the surveillance database is incomplete and there is high uncertainty as to the real prevalence of disease due to the problem of underreporting by farmers (3, 4). Furthermore, the farmers’ awareness of sanitary risk and the way in which they make animal health decisions are often associated with other multiple constraints of an economic, sociological, or cultural nature that do not always favor vaccination as a priority strategy (16). Some authors also mention that studies concerning the farmer’s perception of the socioeconomic impacts of animal diseases are highly relevant in the implementation of disease control strategies (5, 8, 17).

This study aims to use a qualitative method to describe the perception of farmers from South Vietnam regarding vaccination strategies to control FMD. Decisions relative to a given subject are often influenced by socioeconomic factors. A decision is always made according to the perception of the subject (16). Therefore, understanding the perception of farmers is considered critical for the development of a feasible vaccination strategy. The Q methodology—a sociological approach—is a qualitative method used to analyze the subjectivity of individuals faced with a common situation (18). It helps to identify trends and convergences of opinions and patterns within social groups and can be very useful for operators that intend to explore and describe subjective opinions about a particular phenomenon. This method is used in research areas, such as policy (18), public health (19–21), and rural sociology (22).

## Materials and Methods

### Study Zone and Population

This study was conducted in Long An and Tay Ninh provinces, in South Vietnam at the border with Cambodia, from June to October, 2014. The geographical choice was based on three criteria: the importance of livestock production, proximity to the Cambodian border, and the importance of animal movements between provinces and countries. These provinces were also selected in agreement with the DAH and the sub-DAH of the two provinces under study.

**Figure 1:**
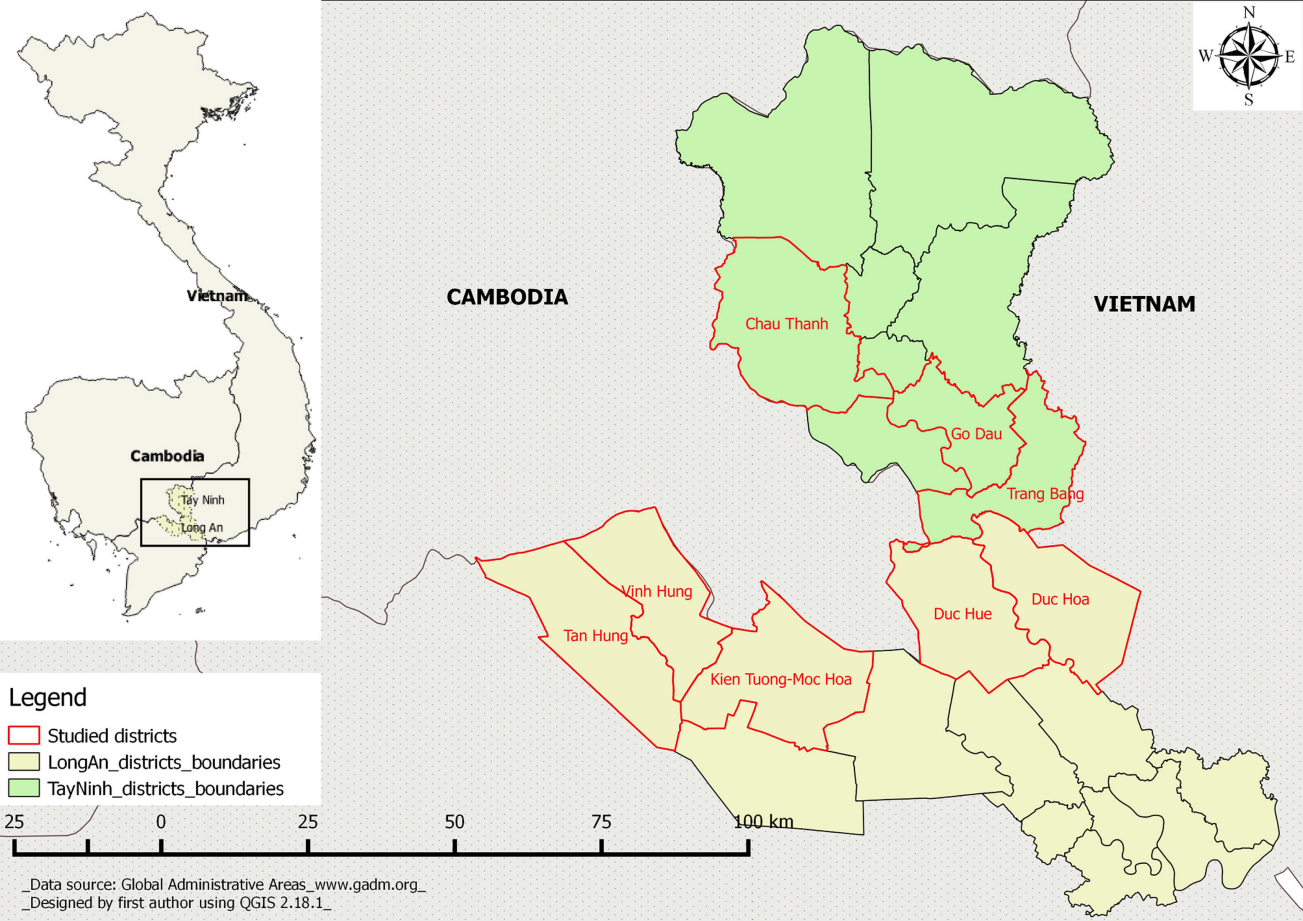
Map of study areas in Long An and Tay Ninh provinces. Yellow: Long An districts; green: Tay Ninh districts; red lines: limited study areas in Long An (Tay Ninh) province.

The first step of our survey was to meet farmers and to record their position on the FMD prevention and control strategy to prepare the Q participatory method. This was performed in five districts of Long An province (Vinh Hung, Tan Hung, Kien Tuong, Duc Hue, and Duc Hoa) and was repeated in three districts of Tay Ninh province (Trang Bang, Go Dau, Chau Thanh). These districts of the two provinces are classified as high-risk zones (11). To record the opinions of different farmers, this study focused on three types of production; dairy cattle, beef cattle, and small pig farms. The number of villages to be visited was calculated for another study done in the same location. The sample size calculations were based on an individual animal prevalence of 30% (9). First, one focus group interview was performed in each selected village. Then, farmers of each production type who displayed willingness to participate in individual interviews were asked to participate in a Q sorting game. Our required sample was 30 villages in each province, i.e., 10 villages in each production type, and at least 10 farmers in each village. The villages were selected from at least three districts in each province to ensure that the study was representative. The number of villages selected from each district was proportional to the districts’ animal population. However, only 54 villages, 27 in each province, contributed to this study due to either incomplete data or a low degree of farmer participation. Each interview was done in the most convenient place for the interviewee (usually at their house) with the participation of two members of the research team. The average duration of interviews was about 1 h. The research team included five people from the Faculty of Animal Science and Veterinary Medicine of Nong Lam University: one veterinary student, two Master’s students, and two professors. The research team members had been trained in participatory methodology by certified trainers 1 month before the start of the field study. Ethical considerations were properly taken into account, as for each interview, each participant signed a written consent to be part of the study. The second step of the survey was to apply the Q method, and this was done in three districts of Tay Ninh province. The study areas are described in Figure 1.

### The Q Methodology

Our survey was conducted in five steps: (i) generation of opinion statements; (ii) selection of the Q set (set of opinion statements); (iii) selection of participants; (iv) Q sorting (sorting of statements by participants) and participant interviews; (v) statistical analysis of each Q sorting (23).

#### Generation of Opinion Statements

Participatory epidemiology (PE) is an emerging field that is based on the use of participatory methods to collect qualitative epidemiological intelligence from community observations, existing veterinary knowledge, and traditional oral history (24). In our survey, PE tools were used to collect initial information from farmers, on their priorities, on FMD prevention and control methods, and on the advantages and limits of vaccination. The PE tools used in this study included semi-structured interviews, using checklists and open questions, focus groups, individual interviews, pair-wise ranking, and flow charts. Further details on the practical aspects of the method’s implementation are described by Mariner (24). The number of participants in each of the 27 focus group interviews varied from 10 to 15 participants. Based on the information collected in the field, an initial list of farmers’ opinions regarding their reasons for vaccinating their animals against FMD, on the perceived advantages and disadvantages of vaccination and other issues related to vaccination in general, was generated. Thanks to the use of PE tools, which allow respondents to express their opinions actively (24), we assumed that relevant information related to farmer’s postures and perceptions was collected.

#### Selection of the Q Set

Based on this list of farmers’ opinions, 46 final statements were produced, representing the spectrum of opinions on vaccination within our population. Four different topics were addressed: (i) farmers’ confidence in vaccination as a preventive method (sense of safety given by the vaccination; control of vaccine production; confidence in suppliers; perception of disease management based on vaccination), (ii) logistics/organization of vaccination in the field (possible constraints due to vaccine practice, type of preferred vaccine, actors delivering the vaccination), (iii) cost of vaccination (affordability for farmers to vaccinate their animals; cost comparison of vaccination with other measures such as treatment, emergency selling), and (iv) impacts of vaccination (on animal productivity and on animals already infected). Detailed statements used in this study are described in Table S1 in Supplementary Material.

#### Participant Selection and Statement Sorting

As mentioned by Brown (18), a Q study requires only a limited number of respondents that is less or equal to the number of statements (18). Based on this concept, a structured sample of respondents, who were relevant to the investigation of FMD vaccination issues, was chosen. Respondents were selected to form a heterogeneous group based on gender (male, female), age (less than or equal to 30 years old, between 30 and 40 years old, between 40 and 50 years old, and more than 50 years old), experience with livestock (less than or equal to 10 years, between 10 and 20 years, more than 20 years), academic level (no school, unknown, primary school, middle school, secondary school, and post-secondary school), production type (beef cattle, dairy cattle, and small pig farm), and location at district level (Trang Bang, Go Dau, and Chau Thanh) in order to capture the points of view of various types of stakeholders. They were contacted individually, several days after their participation in the focus group. We invited 60 individuals to participate in the study. Each respondent was then personally asked to do the Q sorting game. Forty-six cards, representing statements on vaccination were given to the participant while one member of the research team explained the game instructions. The sorting was divided into two phases. First, the farmer was invited to affirm or deny the proposal by freely placing the card on three piles: agree, neutral/ambivalent, and disagree. Then, they continued to put the cards into a quasi-normal grid of 46 boxes. The score given to the statements was proportional to how strongly they agreed or disagreed with them, −3 for strongly in disagreement and +3 for strongly in agreement. When the grid was completed, a discussion with open questions was held, using sentences such as “*you strongly agreed/disagreed with statement n*°…, *why?”*

#### Data Analysis

From the value attributed by the respondents (variables) to the statements (individuals), we created a 46 × 46 matrix. In this matrix, with statements in rows and respondents in columns, the cell values were the scores given by each respondent (25). This first inter-correlation matrix represented the relationship of each Q sort to the other Q sorts (by person), rather than the relationship between statements (19). This correlation matrix was reduced into factors (components) using the principal component analysis (PCA) tool in the “FactoMineR” package (26). Note that the respondents were integrated as variables in the PCA analysis. The first few factors were selected and rotated to obtain a clearer and simpler structure of the data. The usual criteria, according to which the number of factors is selected, include the total amount of variability explained, eigenvalues higher than one, and a compromised solution between complexity and interpretability (25). In our study, factor analysis was done using the “qmethod” (25) package for R. In this step, the three first factors (components) were selected based on the criteria mentioned above and were rotated with the varimax option (maximize of variable) to select the best combination of factors with a cumulative percentage of explained variation over a 40% level-off. Then, the most representative Q sorts for each factor were flagged to select the final combination of factors (most distinguishable perspectives). The criteria for automatic flagging were that 5% of the total Q sort should load distinctly and significantly on each factor with a level of significance set at 99% (*p* < 0.01), which meant that the correlation level was more than 0.38 (2.58*(1/√N)) with *N* = 46 (18, 27). Some Q sorts may be considered as confounding because they loaded highly on more than one factor and thus were not flagged. The normalized *z*-scores that indicate the relationship between statements and factors was a weighted average of the scores given by the flagged Q scores to that statement. The factor scores were calculated by rounding the *z*-scores toward the array of discrete values in the grid. The outcome was three perspectives which were represented by three selected factors at the beginning. These perspectives are a hypothetical Q sort that has been reconstructed from the factor scores (25). Some statements are considered as distinguishing points when the difference between the *z*-scores of a statement in two factors, is statistically significant (based on the SE of differences) (27). When none of the differences between any pair of factors are significant, then the statement is considered a consensus. Automatic flags, statement *z*-scores, and statement factor scores were analyzed using the qmethod package with qflag, qzscores, respectively (25). A Kruskal–Wallis test for non-parametric data was also performed to understand the influence of demographics and characterized variables on the respondent’s contribution to each factor. Interpretation of the results was performed using the ABC model in sociological science (28). According to this model, the attitude of using vaccination as a preventive method can be described according to three main components: an affective component (farmer’s feelings about or valuing of the vaccination), a behavioral component (how the farmers behave toward the vaccination or special tendency or action of farmers who adapt to their attitudes toward vaccination), and a cognitive component (the beliefs about the attitude of using vaccination).

## Results

### Studied Population

**Figure 2:**
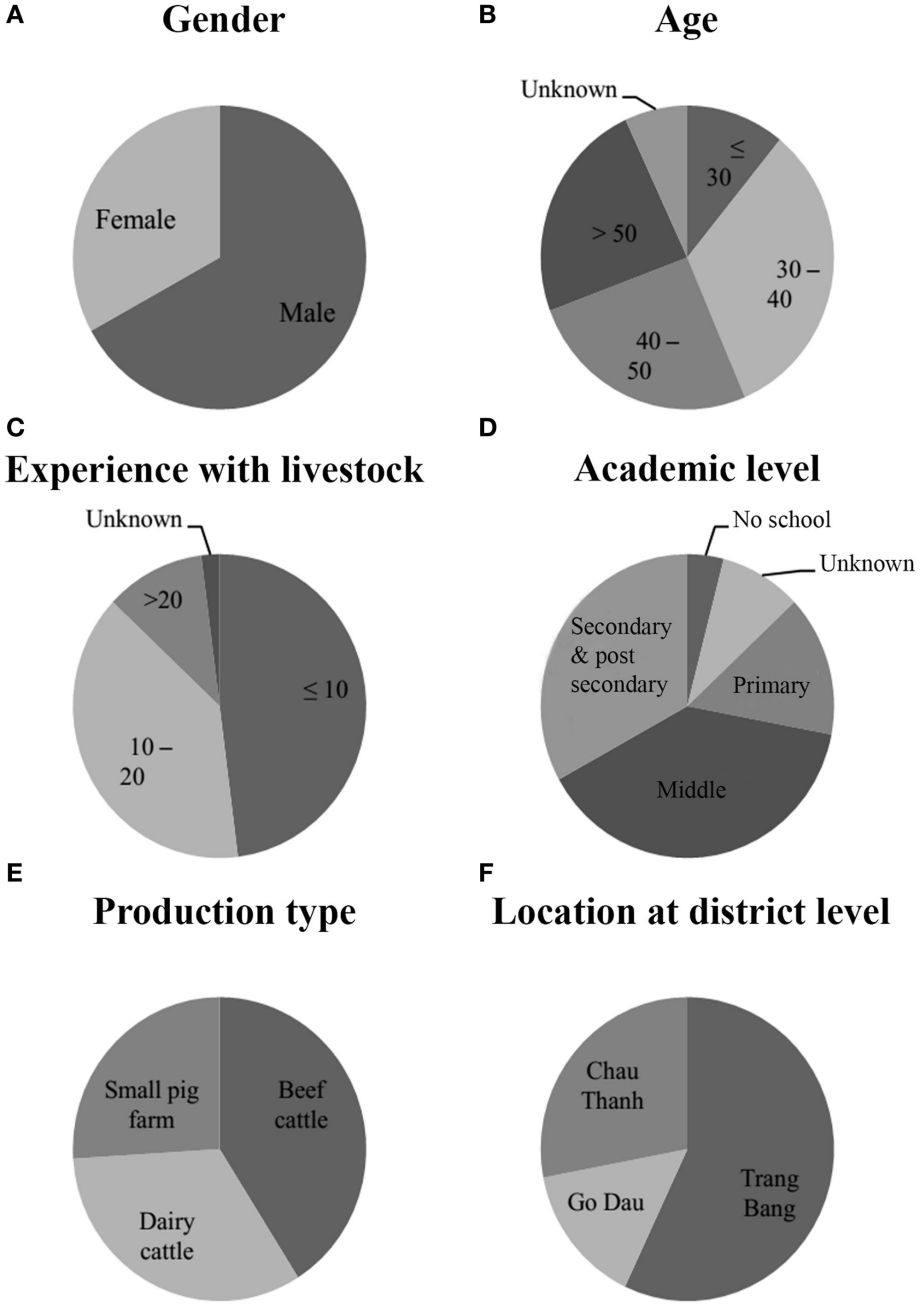
Characterization of the 46 farmers who participated in Q sorting according to variables such as gender **(A)**, age **(B)**, experience with livestock **(C)**, academic level **(D)**, production type **(E)**, and location at district level **(F)**.

From the 60 farmers invited to the meetings held in the 27 villages of the three districts of Tay Ninh province, we were able to identify 46 respondents who fully took part in this study (performed Q sorting game) and included them in our final analysis, in order to match the 46 statements as mentioned by Brown (18). Some of them refused to participate (declined the invitation, too busy, did not understand the game instructions) and others did not follow the game instructions correctly (refused to review their primary results, not providing an explanation for their sorting, and misunderstanding instructions or statements). The studied population is described in Figure 2.

### Q Sorts Analysis

From the PCA results, 10 factors (components) that had an eigenvalue of more than 1.00 were retained. Nevertheless, a full interpretation of the 3 out of 10 factors was carried out in this study, based on the criteria mentioned above (data analysis section) as well as their interpretable nature and the verbatim comments made by the participants. All these factors had an eigenvalue greater than 1.00 and were loaded with at least 5% of the participants. Each factor represented a group of participants who ranked the statements similarly as an indication of a commonly held perception of the issues. The first factor represented 46.2% of the total explained variance. The second and the third factors represented 5.8 and 5.3% of explained variance, respectively. In our analysis, four Q sorts were considered as confounders because they loaded highly on more than one factor and were thus not flagged or not used in the final results. Nevertheless, the three factors selected at the beginning of the analysis were obtained from the PCA calculated with 46 respondents, and so the percentage of variance explained (57.3%) is also calculated for 46 respondents. The remaining 42.7% of the total variance could not be explained by a single factor using the verbatim comments made by the participants, implying that some participants have individual perceptions that cannot be grouped into a single factor.

### Factor Array

Factor analysis was performed on the three selected factors mentioned above, named discourse A, B, and C, respectively. The factor scores (normalized *z*-scores) indicate the pattern of statements that is common to the persons loading on the factor. The most positive values are the statements that the groups strongly agreed with and the most negative values are the statements that the group strongly disagreed with. The summary of statements, scoring for the three factors A, B, and C is presented in Table 1 and Table S1 in Supplementary Material. Table 1 summarizes the perspective of three different groups in grid form where each statement was classified according to their score for each factor. In fact, the result of 46 Q sorts for 46 individuals was generated into three Q sorts of three groups for interpretation. The grid also demonstrated the point of interests for each group through the computed score given by respondents (statements having the scores of ±2 or ±3). Some areas of consensus and disagreement were identified among all the factors, and some statements were identified as distinguishing elements. The list of 46 statements used in this study, shown in Table S1 in Supplementary Material, helped to interpret Table 1. In the description of the different factors, the two numbers in brackets indicate the statement’s number and its score. For example, (stat.19: +3) meaning that statement 19 obtained a positive score of 3.

**Table 1:** Summary of statement scoring for three discourses confidence, belief, and challenge according to the factor analysis.

Discourse confidence							Discourse belief							Discourse challenge						
***−3***	***–2***	***–1***	***0***	***1***	***2***	***3***	***–3***	***–2***	***–1***	***0***	***1***	***2***	***3***	***–3***	***–2***	***–1***	***0***	***1***	***2***	***3***
***20***	**7**	8	4	***6***	**5**	**1**	8	17	**7**	**13**	4	2	**1**	4	8	**7**	2	15	**5**	**1**
32	***21***	17	11	9	12	2	18	***20***	11	**16**	***6***	10	3	18	11	**13**	9	***20***	***6***	3
41	23	18	**13**	10	**14**	3	23	***21***	22	***25***	9	**14**	**5**	41	17	19	12	22	10	23
42	27	***25***	15	22	**24**	19	32	33	27	**34**	12	15	19	42	***25***	27	**16**	28	**14**	**24**
	31	**34**	**16**	30	**29**			40	30	35	26	**24**			31	30	26	33	***21***	
	33	35	26	***37***	44			41	39	36	28	**29**			***38***	**34**	32	***37***	**29**	
		39	28	***38***					43	***37***	31					35	36	44		
		43	36	45					46	***38***	45					46	39	45		
			40							42							40			
			46							44							43			

*Red numbers: score number [with statistically different score values (*p* < 0.05) in one factor compared to the two others]; black number: statement’s number from 1 to 46; bold and underlined numbers: consensus statements; bold italic numbers in shade cells: distinguishing statements*.

### Definition of Three Factors

The three main opinions (i.e., attitudes) belonging to three factors are hereafter referred to as discourses, as is customary in the literature. Discourse A represents the type of farmers who frequently use vaccination because they think that vaccination is an effective tool in disease prevention. We decided to label this discourse “Confidence.” Discourse B includes farmers who also consider vaccination to be a very effective prevention measure but who have a different opinion of vaccination practice (linked to trust in the veterinarian) compared to the group of discourse A. Thus, we decided to label this discourse “Belief.” Discourse C highlights a different opinion on disease management. We decided to label this discourse “Challenge.”

### Discourse A—Confidence

Twenty-four participants contributed to discourse A. According to the results of the Kruskall–Wallis test, no variable (gender, age, experience with livestock, academic level, production type, and location at district level) shows a significant difference in this discourse (Table 2). This means that discourse A is the point of view of a heterogeneous group. Certain main perceptions dominate discourse A. First, the participants appreciate the vaccination because it helps to reduce the farmers’ stress. In fact, they feel that they would suffer from stress if their animals were not vaccinated (stat. 2: +3). In this discourse, farmers consistently declare that they choose to use the FMD vaccine (stat. 19: +3; 20: −3). Their active involvement in the vaccination program is also demonstrated by the fact that the farmers’ decision to vaccinate is not usually influenced by traders (stat. 27: −2), and they have a good comprehension of the vaccination process (sourcing good quality vaccines, administering vaccines to their animals) (stat. 12: +2). Along the same lines, farmers consider that vaccination is an important method of prevention as compared to other husbandry practices (feeding, accommodation) (stat. 32: −3), although they also highlight the need for alternative methods, such as disinfection or quarantine (stat. 31: −2). Finally, in this discourse, farmers are aware of the impact of vaccination on animal productivity (stat. 44: +2).

**Table 2:** Summary of Kruskal–Wallis test for variable analysis.

Variable	Discourse confidence	Discourse belief	Discourse challenge
Gender	ns	ns	*
Age	ns	ns	ns
Experience with livestock	ns	*	ns
Academic level	ns	ns	*
Production type	ns	**	*
Location at district level	ns	ns	ns

p-value, ns, non-significant (p > 0,05); *, significant at 95% (p < 0,05); **, significant at 99% (p < 0,01).

### Discourse B—Belief

Discourse B clearly outlines certain perceptions that differ from discourse A and presents the points of view voiced by 12 participants. The discourse B group of participants is influenced by two variables: livestock experience in years and the production types (Table 2). Participants within this discourse are mainly cattle farmers (including dairy cow and beef) and have more than 10 years of experience in livestock production. Similar to discourse A, farmers in discourse B consider that adequate vaccination practices are needed to achieve a good level of protection (stat. 3: +3). They think that vaccines and services delivered by the governmental veterinary services are always very efficient in controlling diseases (stat. 10: +2; 14: +2) and that the quality of a vaccine is subject to its price (stat. 15: +2). Finally, these participants share the same approach: they systematically decide to vaccinate their animals against FMD, even if there is no outbreak close to their village (stat. 23: −3) because they are located in a high-risk zone. However, these farmers, unlike the ones from the discourse A, prefer to have their animal vaccinated by a veterinarian rather than doing it themselves (stat.40: −2).

### Discourse C—Challenge

Discourse C represents the perception of six participants. This group of backyard farmers, including five women and one man, keep on average 23 pigs (4 pig farmers) or 16 beef cattle on their farms (2 cattle farmers) and have, on average, 15 years of experience with livestock (Table S2 in Supplementary Material). Statistically, discourse C is influenced by the three following variables: female gender, pig production, and primary school academic level (Table 2). The first element dominating discourse C is their perception of vaccine effectiveness. They claim to vaccinate their animals to protect them from surrounding herds (stat. 6: +2) and also refuse to introduce a new animal if they do not know its vaccination status (stat. 4: −3). For them, vaccination is not 100% effective, so they need to combine the two control measures to minimize the probability of introducing the disease in the herd. In this discourse, participants consider that the vaccines proposed by veterinarians are well-conserved (stat. 17: −2) and they have more confidence in these vaccines than in the ones they can buy elsewhere (stat. 11: −2). One of the most important perceptions distinguishing this discourse relates to the participants’ opinions on disease management. According to their discourse, they do not always vaccinate their animals (stat. 21: +2). They only vaccinate when there is an outbreak close to their village (stat. 23: +3). Moreover, their decision is not influenced by their neighbors’ behavior (stat. 25: −2) or by the cost of vaccination (stat. 41: −3). Finally, they do not like to buy multi-dose vials as these are not suited to their production scale (stat. 38: −2).

### Consensus and Distinguishing Points

Several consensual points were found across the three discourses. All of the farmers in the study zone felt more secure after taking part in the vaccination campaign (stat. 1 and 5); they make vaccination decisions themselves without being influenced by their neighbor’s decisions or by traders (stat. 24); they believe in the veterinary information that they receive on disease risk (stat. 29); and they also perceive that vaccination is cheaper than treatment (stat. 41) and vaccines provided by governmental authorities are of good quality (stat. 7 and 14). However, there were several points of disagreement between the discourses. Some farmers (discourse “Challenge”) believe that they do not need to vaccinate their animals every year (stat.21) if the housing and feeding conditions are right (stat.32, 33) or if there is no outbreak in neighboring villages (stat 23). Also, some participants of this discourse claim that they have never used vaccines in their herd (stat 20), because they have never experienced this disease before. The preferred type of vaccine to purchase (individually or multi-dose) differs between discourses (stat. 37, 38).

## Discussion

### The Farmer’s Perception of FMD Vaccination

#### Effectiveness of Vaccination

Some advantages of vaccination are recognized by the farmers, such as the contribution to stress management and savings made, thanks to the vaccination rather than the more costly treatment option and the compensation received in the case of infection within a vaccinated herd. These benefits are also clearly justified by some participants who had the experience of affected herds before using vaccination. The farmers’ strong belief in governmental vaccination programmes was clearly demonstrated. This can be explained first by the vaccine quality control implemented by governmental authorities. Second, by the fact that the epidemiological situation of FMD is supervised throughout surveillance (serologic status, outbreak investigation, post-vaccination monitoring, vaccine matching with the help of regional and worldwide FMD reference laboratories) (11, 15) that provide regular recommendations on the strains of vaccine to be used for each province. Therefore, during 2011–2014, thanks to the help of the vaccination program, only two outbreaks were recorded in Tay Ninh province (15).

All of the farmers in the study zone perceive that the cost of vaccination is cheaper than that of treatment, for several reasons. First, the vaccines used by farmers who participate in vaccination campaigns are provided by the government free of charge. Participants only pay for the cost of veterinary work, from 0.09 to 0.18 USD per injection in pigs and cattle (11). Otherwise, they can buy the vaccine themselves at the price of 0.76 USD for a monovalent dose and 1.08 USD for a bivalent vaccine (official vaccination price from sub-DAH of Long An province). For example, for each head of cattle that is vaccinated twice yearly, the farmer must pay around 0.36–2.16 USD per head of cattle. Whereas, for the treatment of FMD, veterinary services (disinfection, consultation, medicines) are required over a duration of at least 3–5 days and can cost around 13.5–15.5 USD per head of cattle (personal communication).

#### Choice of Vaccine Type

The preferred type of vaccine doses (individual or multi-dose) depends on the discourse (stat. 37, 38). Some prefer individual doses for immediate use because of their small herds and difficulties regarding preservation. Others like to use multi-dose vials because they have big herds and vaccine preservation is not an issue for them. Then, there is a share of the population that uses neither individual doses, due to traceability problems, nor multi-doses due to the cost of the vaccine; they opt for other prevention methods (hygiene, disinfection, good husbandry) instead. Only vials containing 25 doses are available; however, farmers can order individual doses from private veterinary practitioners if needed. Each dose is contained in a single syringe and must be used immediately after purchasing.

#### Decision-making and Trends

The fact that the farmer’s vaccination decision is not influenced by other stakeholders (stat. 24) illustrates one of the psychological traits of Vietnamese farmers. According to Ref. (29), their production is small-scale and scattered, they have a traditional lifestyle, tend to rely on experience, and are reluctant to innovate. As they are influenced by small-scale production, they tend to rely on their accumulated experiences to guide their decisions on significant concerns. Our findings differ to those reported by Young et al. (30) in Lao, where traders indicated that they prefer to buy vaccinated animals to protect their investment (30) and might be influenced by other farmers’ decisions. Our findings raise a question as to the sustainability of farmers’ vaccination practices if they no longer receive governmental support. Dairy cow farmers will certainly continue to buy and use vaccines as the disease is a direct threat to their daily income from milk. However, for beef cattle and pig farmers, the maintenance of FMD vaccination is uncertain, as they can sell incubated or recovered animals that are free of clinical signs to traders since there is no stamp-out method for affected animals (15). This trend may be confirmed by the vaccination approach adopted by discourse Challenge farmers; the latter think that they do not need to vaccinate their animals every year (stat. 21) if the housing and feeding conditions are good or if there is no presence of outbreak in surrounding farms (stat. 23). Also, a minority share of participants indicated that they never use vaccines in their herd (stat. 20) because they had never been affected by FMD. Therefore, some farmers do not consider vaccination to be the first choice among prevention methods.

Farmers from discourse Confidence and Belief fully vaccinate their animals, either themselves (Confidence) or with the help of a veterinarian (Belief). This difference mainly lies in the trust given to the veterinarian depending on the different types of farmers. It seems that dairy farmers strongly believe that veterinarians can contaminate their herds through their visit, while beef cattle farmers place more trust in the veterinarians. Therefore, dairy farmers prefer to organize the vaccination by themselves, i.e., sourcing good quality vaccines and administering them to animals, to ensure the vaccination’s effectiveness (stat. 12: +2). In contrast, beef farmers prefer to have their animals vaccinated by the veterinarian (stat. 40: −2). When there are difficulties linked to the delivery of the vaccine, dairy farmers show greater motivation to find other sources of vaccine than beef cattle farmers. This is because the vaccination for the latter group (product supply and injection) is mainly done with the help of a veterinarian (direct observation and in-depth discussion).

Rational-choice and risk analysis theories can provide a valuable contribution to understanding the vaccination choices made by farmers. The rational-choice theory, derived from the fields of philosophy, anthropology, and economics, explains that an individual always acts intentionally, evaluating options and seeking to use resources rationally to achieve the highest possible cost/benefit ratio (31). This means that before deciding on a certain action, individuals always weigh up the balance between cost and benefits, if the cost is equal to or less than the benefits they will engage in the action (as did discourse Confidence and Belief farmers), but if the cost of the action outweighs its benefits, they will not engage in the action (discourse Challenge). Although the cost of vaccination is considered to be inexpensive, farmers who are classified as having medium or low incomes (32, 33), feel that avoiding this expense will benefit them, especially for pig farmers who do not receive government compensation for vaccination. Moreover, the low mortality among affected animals supports their decision to refuse vaccination.

The risk analysis theory can also be used to explain farmers’ choices. According to this theory, farmers consider two elements when evaluating the risk of infection: the probability of being infected and the consequences of infection (34). For cattle farmers, the likelihood of infection is high, since sero-prevalence in cattle in hotspot areas (including our study side) is nearly 30% (9). Moreover, the different consequences can be an interesting variable to explain the distinction between dairy cow and beef cattle farmers’ motivation to vaccinate. For dairy cow farmers, their income depends on the volume of milk that they sell every day. To sell milk to milk collectors, they must produce a certificate of vaccination against infectious diseases, including FMD, to prove that their animals are well-protected. This forces them to vaccinate their animals every 6 months. An FMD outbreak will cause them to lose part of their income, although they will be able to continue selling their product. However, if certification is lacking or has expired upon the collector’s control, they will immediately be banned from selling their milk. In this case, farmers will have to sell off their valuable dairy cows at the price of basic beef cattle to survive; they, therefore, decide to vaccinate their animals. Income from beef cattle is raised when the animals are sold after several months or years of fattening. An affected animal with FMD can be symptomatically cured, with folk remedies made by themselves from experience, i.e., cashew nut (*Anacardium occidentale)*, false daisy *(Eclipta prostrata)* or found in traditional medicine stores (personal communication), and can then be sold at the usual price after treatment. Therefore, the disease has little impact on farmers. This explains why vaccination is implemented by a lower percentage of beef farmers than dairy cow farmers. For the remaining farmers (discourse Challenge), the probability of disease outbreak is lower, with moderate consequences, thanks to the possibility of emergency sales of infected/dead animals at lower prices than the usual price; they, therefore, choose not to vaccinate and sell their animals if needed. Farmers might underestimate the consequences of FMD in their herds because they have never experienced an outbreak. In fact, it is reported that consequences for pig farmers are substantial because of the high mortality caused by FMD, especially in piglets (almost 100%) (35). With better information, we could get farmers from this group to vaccinate more, allowing them to benefit from increased revenues and decreased levels of stress when an outbreak occurs in their zone. Actually, in this hypothetical situation, a vaccinated animal (assuming that the animal is fully protected, thanks to vaccine) could be sold at a normal price while the non-vaccinated animals of neighbor farms could only be sold at half the price or less. Farmers with vaccinated animals could maintain their revenue and avoid the stress experienced by owners of non-vaccinated animals who have to find a way to sell their animals as quickly as possible in the event of an outbreak.

### Discussion on PE and Q Methodology

For the PE approach, participants of the focus group interviews were usually invited by the commune’s local veterinarian or by the village chief, meaning that the objective of the study must be well understood by these the main actors. An undesired consequence, which may form a bias in our study, is the lack of representativeness of our sample. In fact, the majority of participants have a close relationship with these key persons (clients, family members, neighbors, and members of a particular group), and this may have modified the opinions expressed on certain sites. The problem of over-representativeness can be observed in discourse Confidence. Organizing more than one focus group per village would help to solve this issue, although this is not possible in a time-limited survey. Another potential bias related to our studied population is the selection of only two volunteers per village to undertake the Q sorting; also, these two volunteers were not always the ones identified by the randomized selection. This constraint might be an obstacle to the discovery and understanding of certain perceptions of the farmers who had been randomly selected in advance but who declined to participate in the game.

Sociological methods such as Q methodology have been widely applied in policy, public health, rural sociology but have been remained very limited in the field of veterinary sciences. This method may, therefore, be considered as an innovative approach in this field. During the implementation of our survey, the veterinary authorities questioned the feasibility and effectiveness of these tools. However, to assess the validity of our findings, data were triangulated and confirmed with information collected during each interview with the help of open-end questions. The collection of information from a heterogeneous group of farmers in 30 randomized villages, located in different sites, ensured that our results were representative. Q methodology facilitated the active participation of respondents as they were free to classify statements within a grid and to explain the reasons for their choices during open follow-up interviews. These advantages helped to maintain the study’s objectivity. The logical nature of a particular viewpoint was easy to check after the Q sorting process with open-end questions. This method also forced people to rank their preferences using a pre-defined grid score (with negative and positive points). Thus, the researcher could fully understand the point of interest as well as the source of their agreement or disagreement of the prioritized issues. During the data analysis process, each Q statement was sorted relatively to all other statements; this method, therefore, conserved the universal nature of a viewpoint better than surveying methods. Regarding practicability and simplicity, the strong point of this methodology is that it only required simple materials and the participation of a small number of respondents (22). However, this method could also be the source of biases. First, this exercise lasted more than 1 h for each participant, which was long and might have made them feel uncomfortable. As a consequence, the responses to the open-end questions at the end of this exercise, explaining their choices, were very short. Second, due to field constraints, the statement sorting activity was organized after a focus group interview on the topic of prevention and control methods of critical diseases in their animals. As the participants were aware of the research objectives before doing the game, it gave the impression that they were encouraged to express a favorable opinion on vaccination, which did not always reflect their original opinion. A bias might also have been being introduced due to the type of interviewer, as the latter was related to vet services to avoid any possible conflicts in the future. Finally, some participants complained that certain statements were organized in a contradictory or complicated manner, making them difficult to understand. Indeed, some of the statements were too difficult for the farmers; this concerned virus circulation, virus strains, the concept of emergency vaccination, etc. These points should be reviewed for further research.

### Recommendation

It is important to note that a part of the studied population does not consider vaccination to be the first choice among preventive methods. This finding raises the question of how to improve the active participation of farmers in the vaccination strategy against FMD to eradicate the disease from Vietnam (cf. farmers’ challenges found in our study). Regular awareness raising is an important tool to encourage active participation and maintain the farmers’ motivation to vaccinate (36). It would seem that highly experienced beef farmers and women who raise a small number of pigs are the main actors who could benefit from a change in behavior and attitude. A few key messages that we recommend to be conveyed are listed below: (i) selling infected animals is forbidden by policy; (ii) vaccination certification facilitates trade and compensation from the government if a vaccinated animal is declared infected; (iii) district veterinary centers are safe places to buy vaccines; (iv) compensation is available only once per year through the government support scheme and the effect of vaccination lasts only 6 months, so farmers need to buy vaccines themselves and vaccinate their animals twice a year; (v) vaccinating only when there is an outbreak close to the village is often ineffective due to the fast transmission of the virus; and (vi) good husbandry and disinfection are not enough to protect animals from infection. A good way for the veterinary services to prove the advantages of vaccination versus other control methods, such as the treatment or sale of sick animals, would be to implement simple cost–benefit analyses at farm level and to communicate the results. Moreover, a clear message from the authorities on the risk of FMD in pigs would help people to make appropriate choices to achieve the eradication of the disease. Other recommendations for vaccine suppliers could be to develop smaller packages, such as only 5 or 10 doses per vial, to tailor their products to the needs of small-scale production.

## Conclusion

These results highlighted the fact that farmers in our study zone are aware of the objective of vaccination, its role and its value in preventing disease. Prevention by vaccination was also understood to be cheaper than treatment costs and vaccines provided by governmental authorities were perceived as being of good quality. However, a minor part of the population expressed doubts regarding vaccination as a prevention method. These results illustrated critical elements that influence the acceptability of the FMD programme by farmers in Vietnam and allowed certain recommendations to be developed on how to improve farmer involvement in national FMD control and prevention programmes. Their participation is critical to maintain a high vaccine coverage of populations and to ensure the success of the national program. Further research is required to better understand farmers’ perceptions and how they interact with other stakeholders involved in the vaccination campaign.

## Ethics Statement

Ethical considerations have been properly taken in consideration, as for each interview, each participant has signed a written consent to be part of this study. The data were also analyzed anonymously and reported. Our study is approved by the local authorities (sub-Department of Animal Health of Long An and Tay Ninh). A confirmation letter of this approval has been sent to the journal editor upon request. In Vietnam, this study was considered as a common study on animal health, and therefore, no ethical committee is provided by the national authorities.

## Author Contributions

DT, AB, and FG designed the study, contributed to the analyses, and drafted the manuscript. BT, NN, and MP designed the data collection instrument and drafted the manuscript. MP and SB reviewed the results and drafted the manuscript. The manuscript has been read and approved by all authors.

## Conflict of Interest Statement

The authors declare that the research was conducted in the absence of any commercial or financial relationships that could be construed as a potential conflict of interest.

## Abbreviations

FMD, foot-and-mouth disease; PCA, principal component analysis; DAH, Department of Animal Health; MARD, Ministry of Agriculture and Rural Development; PE, participatory epidemiology; Stat., statement [only used with number to indicate the particular statement(s) that is (are) mentioning in paragraph].
