# Supplementary material for: A Q Method Approach to Evaluating Farmers’ Perceptions of Foot-and-Mouth Disease Vaccination in Vietnam
Source: Front Vet Sci. 2017 Jun 26;4:95. doi: 10.3389/fvets.2017.00095 (PMC5483627; doi:10.3389/fvets.2017.00095)
Supplement: Supplementary file 1 [file Table_1.DOCX]

**Supplementary Information**

**Supplementary table 1. List of statements used in this study and the statement factor scores^1^ and statement z-scores^2^ (in parenthesis) according to each factor/ discourse after Q-sort analysis, including consensus statements**

| **Statement** | **Factor/Discourse** | | | **Consensus statements** |
| --- | --- | --- | --- | --- |
|  | **1** | **2** | **3** |  |
| **1.1. Stress management** | | | | |
| 1. I feel more secure after my animals are vaccinated against FMD | 3 (1.87) | 3(1.94) | 3(1.5) | X |
| 2. I am stressed if I do not vaccinate my animals against FMD | 3(1.58) | 2(1.46) | 0(-0.03) |  |
| 3. When the FMD vaccination is well done, my animals are completely protected against the disease | 3(1.7) | 3(1.96) | 3(1.48) |  |
| 4. I can introduce a new animal without fear of FMD if my animals are vaccinated against the disease | 0(0.03) | 1(0.32) | -3(-1.79) |  |
| 5. I vaccinate my animals to protect them from FMD | 2(1.47) | 3(1.65) | 2(1.23) | X |
| 6. I vaccinate to protect other herds from FMD | 1(0.7) | 1(0.32) | 2(1.41) |  |
| **1.2. Product control/ supplier confidence** | | | | |
| 7. I have already refused vaccination against FMD because I thought that the vaccine was bad | -2(-0.93) | -1(-0.78) | -1(-0.61) | X |
| 8. FMD vaccines produced in China are of good quality | -1(-0.78) | -3(-1.24) | -2(-0.88) |  |
| 9. FMD vaccines that come from the vet shop are of good quality | 1(0.24) | 1(0.68) | 0(0.17) |  |
| 10. FMD vaccines used by veterinarians are of good quality | 1(0.9) | 2(1.59) | 2(1.23) |  |
| 11. I have more confidence in a vaccine that I bought myself than the vaccine provided by the veterinarian. | 0(-0.23) | -1(-0.8) | -2(-1.14) |  |
| 12. I understand whom to ask and how to organize the vaccination of my animals against FMD with good products | 2(0.93) | 1(0.81) | 0(0.04) |  |
| 13. It is easy to identify whether an FMD vaccine is produced locally, in China or another country | 0(-0.12) | 0(-0.19) | -1(-0.49) | X |
| 14. The effectiveness of the product depends on the identity of individuals (place of supply) who provide me with the FMD vaccine | 2(1.01) | 2(1.07) | 2(0.97) | X |
| 15. I believe that the higher the quality of the vaccine, the more expensive it is. | 0(0.11) | 2(1.28) | 1(0.54) |  |
| 16. The FMD vaccine used by veterinarians is not specific to the virus circulating | 0(-0.09) | 0(-0.15) | 0(-0.3) | X |
| 17. The FMD vaccines used by veterinarians are not well preserved | -1(-0.36) | -2(-1.1) | -2(-1.16) |  |
| 18. The FMD vaccines used by veterinarians are counterfeit | -1(-0.43) | -3(-1.48) | -3(-1.65) |  |
| **1.3. Perception/ disease management** | | | | |
| 19. I always have my animals vaccinated against FMD | 3(1.77) | 3(1.63) | -1(0.86) |  |
| 20. I never vaccinate my animals against FMD | -3(-1.9) | -2(-1.18) | 1(0.91) |  |
| 21. In certain past years, I did not vaccinate my animals against FMD | -2(-1.31) | -2(-0.88) | 2(1.44) |  |
| 22. I vaccinate part of my herd against FMD | 1(0.56) | -1(-0.56) | 1(0.84) |  |
| 23. I vaccinate only when there is an FMD outbreak near my village | -2(-1.43) | -3(-1.27) | 3(1.79) |  |
| 24. I take the decision to vaccinate alone (individually) | 2(1.22) | 2(1.34) | 3(1.52) | X |
| 25. I take the decision to vaccinate in consultation with my neighbors | -1(-0.59) | 0(0.15) | -2(-1.16) |  |
| 26. I take the decision to vaccinate in consultation with my family | 0(-0.34) | 1(0.89) | 0(-0.08) |  |
| 27. My decision to vaccinate is influenced by traders | -2(-1.08) | -1(-0.57) | -1(-0.66) |  |
| 28. My decision to vaccinate is influenced by the veterinarian’s messages | 0(0.11) | 1(0.47) | 1(0.34) |  |
| 29. I believe that the diseases for which veterinarians propose vaccines are diseases that my animals are at risk of being contaminated with | 2(1.26) | 2(1.18) | 2(1.31) | X |
| 30. Veterinarians can contaminate my herd with FMD during vaccination | 1(0.77) | -1(-0.75) | -1(-0.53) |  |
| 31. If my animals are vaccinated against FMD, I would not need to protect my animals with other methods (disinfection, quarantine) | -2(-1.23) | 1(0.45) | -2(-0.94) |  |
| 32. If I keep my animals in good condition (good food, good housing), I do not need to vaccinate them against FMD | -3(-1.47) | -3(-1.35) | 0(-0.36) |  |
| 33. If I properly disinfect my buildings, I do not need to vaccinate my animals against FMD | -2(-0.88) | -2(-1.12) | 1(0.39) |  |
| 34. For a pregnant cow or a calf, we must inject half of the normal dose | -1(-0.48) | 0(-0.5) | -1(-0.78) | X |
| **2. Logistics/ Organization of vaccination** | | | | |
| 35. Vaccination of my animals against FMD causes more work (constraints) | -1(-0.82) | 0(-0.07) | -1(-0.61) |  |
| 36. The timing proposed by the veterinary services for vaccination against FMD do not suit my calendar | 0(-0.2) | 0(-0.43) | 0(0.12) |  |
| 37. I prefer to buy vaccines in single doses | 1(0.92) | 0(-0.02) | 1(0.46) |  |
| 38. I prefer to buy the vaccines in a multi-dose vial | 1(0.27) | 0(-0.12) | -2(-0.94) |  |
| 39. Asking a veterinarian to give the injections costs me a lot more | -1(-0.36) | -1(-0.62) | 0(-0.08) |  |
| 40. I prefer to vaccinate my animals myself rather than to let the veterinarian do it | 0(-0.07) | -2(-1.16) | 0(-0.42) |  |
| **3. Vaccination cost** | | | | |
| 41. I think the cost of treatment is cheaper than vaccination | -3(-1.49) | -2(-1.09) | -3(-1.43) |  |
| 42. I think the loss of money paid by the trader when buying a sick animal infected with FMD is lower than the cost of vaccination | -3(-1.44) | 0(-0.41) | -3(-1.84) |  |
| 43. I think the cost of vaccination against FMD in my budget is too high | -1(-0.85) | -1(-0.66) | 0(0.08) |  |
| **4. Vaccination impact** | | | | |
| 44. Vaccination against FMD decreases animal productivity (weight, milk) | 2(1.07) | 0(-0.17) | 1(0.73) |  |
| 45. Vaccination of pregnant animals against FMD causes abortions | 1(0.26) | 1(0.21) | 1(0.84) |  |
| 46. ​​Vaccination of animals that are already infected with FMD causes sudden death | 0(0.14) | -1(-0.72) | -1(-0.62) |  |

^1^ Statement factor score (other name: rang value, round value): the scores rounded to match the array of discrete values in the distribution of predefined grid score (-3 to +3).

^2^ Statement z-score: (other name: non-round value): the weighted average value of each statement for each factor.

**Supplementary table 2. Summary of demographic and characterised variables of the respondents who contributed to three discourses A, B, and C**

| **Variable** | **Discourse A** | **Discourse B** | **Discourse C** |
| --- | --- | --- | --- |
| **Gender**  + Male  + Female | 18  6 | 10  2 | 1  5 |
| **Age**  + Under 30  + 30 – 40  + 40 – 50  + More than 50  + Unknown | 3  10  6  5  0 | 1  3  2  3  3 | 0  1  3  2  0 |
| **Experience with livestock**  + Under 10 years  + 10 – 20 years  + More than 20 years  + Unknown | 12  12  0  0 | 5  2  4  1 | 2  3  1  0 |
| **Academic level**  + No school  + Primary school  + Middle school  + Secondary & post secondary school  + Unknown | 1  2  12  8  1 | 1  0  4  4  3 | 0  4  1  1  0 |
| **Production type**  + Beef cattle  + Dairy cattle  + Small pig farm | 7  11  6 | 7  3  2 | 2  0  4 |
| **Location at district level**  + Trang Bang  + Go Dau  + Chau Thanh | 19  2  3 | 4  3  5 | 1  2  3 |
